# Variations in corticosteroid/anesthetic injections for painful shoulder conditions: comparisons among orthopaedic surgeons, rheumatologists, and physical medicine and primary-care physicians

**DOI:** 10.1186/1471-2474-8-63

**Published:** 2007-07-06

**Authors:** John G Skedros, Kenneth J Hunt, Todd C Pitts

**Affiliations:** 1 Utah Bone and Joint Center, Salt Lake City, Utah, USA; 2 University of Utah Department of Orthopaedic Surgery, Salt Lake City, Utah, USA

## Abstract

**Background:**

Variations in corticosteroid/anesthetic doses for injecting shoulder conditions were examined among orthopaedic surgeons, rheumatologists, and primary-care sports medicine (PCSMs) and physical medicine and rehabilitation (PMRs) physicians to provide data needed for documenting inter-group differences for establishing uniform injection guidelines.

**Methods:**

264 surveys, sent to these physicians in our tri-state area of the western United States, addressed corticosteroid/anesthetic doses and types used for subacromial impingement, degenerative glenohumeral and acromioclavicular arthritis, biceps tendinitis, and peri-scapular trigger points. They were asked about preferences regarding: 1) fluorinated vs. non-fluorinated corticosteroids, 2) acetate vs. phosphate types, 3) patient age, and 4) adjustments for special considerations including young athletes and diabetics.

**Results:**

169 (64% response rate, RR) surveys were returned: 105/163 orthopaedic surgeons (64%RR), 44/77 PCSMs/PMRs (57%RR), 20/24 rheumatologists (83%RR). Although corticosteroid doses do not differ significantly between specialties (p > 0.3), anesthetic volumes show broad variations, with surgeons using larger volumes. Although 29% of PCSMs/PMRs, 44% rheumatologists, and 41% surgeons exceed "recommended" doses for the acromioclavicular joint, >98% were within recommendations for the subacromial bursa and glenohumeral joint. Depo-Medrol^® ^(methylprednisolone acetate) and Kenalog^® ^(triamcinolone acetonide) are most commonly used. More rheumatologists (80%) were aware that there are acetate and phosphate types of corticosteroids as compared to PCSMs/PMRs (76%) and orthopaedists (60%). However, relatively fewer rheumatologists (25%) than PCSMs/PMRs (32%) or orthopaedists (32%) knew that phosphate types are more soluble. Fluorinated corticosteroids, which can be deleterious to soft tissues, were used with these frequencies for the biceps sheath: 17% rheumatologists, 8% PCSMs/PMRs, 37% orthopaedists. Nearly 85% use the same non-fluorinated corticosteroid for all injections; <10% make adjustments for diabetic patients.

**Conclusion:**

Variations between specialists in anesthetic doses suggest that surgeons (who use significantly larger volumes) emphasize determining the percentage of pain attributable to the injected region. Alternatively, this might reflect a more profound knowledge that non-surgeons specialists have of the potentially adverse cardiovascular effects of these agents. Variations between these specialists in corticosteroid/anesthetic doses and/or types, and their use in some special situations (e.g., diabetics), bespeak the need for additional investigations aimed at establishing uniform injection guidelines, and for identifying knowledge deficiencies that warrant advanced education.

## Background

Injectable corticosteroids are commonly used by orthopaedic surgeons, rheumatologists, primary-care physicians and other health-care providers in the treatment of painful shoulder conditions. However, surveys have estimated that 60% to 70% of internists finishing their residency training feel that they need more training in performing these injections [1]. Furthermore, even though corticosteroid injections are commonly used for painful shoulder conditions, there are no uniform guidelines regarding dosage and other aspects of their administration. In order to work towards this goal, baseline information regarding corticosteroid usage is needed. We were not able to locate published data comparing and contrasting surgeon and non-surgeon musculoskeletal specialists who treat painful shoulder conditions with these injections. Additionally, our literature review of studies (including meta-analyses) evaluating the use of corticosteroid injections for painful shoulder conditions show a lack of consensus regarding their dosing and time course of administration [2-12] (Table 1). Among these reviews, we also observed that confusion often arises regarding dosing when making a direct correlation between equivalences and relative potencies of corticosteroids (Tables 2 and 3). This lack of uniform injection guidelines is important because deleterious consequences and other sequelae, both systemic and local, can result from corticosteroid injections, especially from chronic use, large doses, and errant injection (e.g., into a tendon) [13].

**Table 1:** Various recommended corticosteroid dose ranges. Currently Recommended Corticosteroid ("Cortisone") Dose Ranges

**Generic Name **(Tradename)	**Intermediate Joint^a ^(mg) [A-C Joint]**	**Large Joint^a ^(mg) [S-A Bursa and G-H Joint]**	**References**
**Methylprednisolone diacetate **(Depo-Medrol^®^)	-	40–60	Cush and Kavanaugh (2000)^6^
	20–30	40–80	Greene (2001)^7^
	20–40	40–80	Tallia and Cardone (2003)^3^
	-	20–80	Noerdlinger and Fadale (2001)^2^

**Prednisolone tebutate **(Hydeltra-TBA^®^)	5–50^b^	5–50^b^	Cush and Kavanaugh (2000)^6^
	-	10–40	Noerdlinger and Fadale (2001)^2^

**Triamcinolone acetonide **(Kenalog^®^)	5–40^b^	5–40^b^	Cush and Kavanaugh (2000)^6^
	10	20	Saunders (2002)^4^
	-	5–40	Noerdlinger and Fadale (2001)^2^
	10–20	40–80	Moore (2001)^8^

**Triamcinolone hexacetonide **(Aristospan^®^)	5–40^b^	5–40^b^	Cush and Kavanaugh (2000)^6^
	-	10–20	Noerdlinger and Fadale (2001)^2^

**Betamethasone sodium phosphate – Betamethasone acetate **(Celestone Soluspan^®^)	1.5–6^b^	1.5–6^b^	Cush and Kavanaugh (2000)^6^
	1.5–3	6–12	Tallia and Cardone (2003)^3^
	-	6–12	Noerdlinger and Fadale (2001)^2^

^a ^**"Intermediate joints" **= A-C, elbow, wrist, temporal mandibular, and ankle; **"Large joints" **= S-A bursa, G-H, hip, and knee (Source: Current Procedural Terminology (CPT) 2004, American Medical Association, AMA Press)

^b ^The authors listed suggested these general ranges for both intermediate and large joints.

**Table 2:** Common injectable corticosteroids. Common Injectable Corticosteroids ("Cortisones")

				**Recommended Dose Range^c^**	
				
**Solubility/Generic Name**	**Common Trade Name**	**Strength^a ^(mg/cc)**	**Relative Potency^b^**	**Intermediate Joint [A-C Joint] (mg)**	**Large 'Joint' [S-A Bursa and G-H Joint] (mg)**
**Most Soluble**					
* Betamethasone sodium phosphate	Celestone Phosphate^F^	3	25	1.5–3	6–12
					
**Soluble**					
* Dexamethasone sodium phosphate	Decadron^F^	4	25	2–4	7.5–15
Prednisone sodium phosphate	Hydeltrasol	20	4	12.5–25	50–100
					
**Slightly Soluble**					
* Methylprednisolone acetate	Depo-Medrol	20/40/80	5	10–20	40–80
Triamcinolone diacetate	Aristospan Forte^F^	25/40	5	10–20	40–80
Prednisolone tebutate	Hydeltra-TBA	20	4	12.5–25	40–80
					
**Relatively insoluble**					
* Triamcinolone acetonide	Kenalog^F^	10/40	5	10–20	40–80
* Triamcinolone hexacetonide	Aristospan^F^	20	5	10–20	40–80
Hydrocortisone acetate	Hydrocortone	25	1	25–50^d^	100–200^d^
Dexamethasone acetate	Decadron-LA^F^	8	25	2–4	7.5–15
					
**Combination**					
* Betamethasone sodium phosphate-Betamethasone acetate	Celestone Soluspan^F^	6	25	1.5–3	6–12

Chart adapted from: Noerdlinger and Fadale (2001)^2^, Saunders (2002)^4^, Tallia and Cardone (2003)^3^, Axelrod (1976)^10^, and Klippel et al. (2001)^9^.

* The most commonly used cortisones among the physicians surveyed in the present study.

**F **= fluorinated compounds.

**^a ^**Adjustments in reported "cortisone" volumes (doses) in our survey respondents were made in cases where different strengths are available. In these cases the volumes reported in the results section are: 40 mg/cc for Depo-Medrol, Aristospan Forte, and Kenalog; 25 mg/cc for Hydrocortone.

**^b ^**For example, 1 mg Celestone = 5 mg Depo-Medrol = 5 mg Aristospan = 25 mg Hydrocortisone (See Table 3).

**^c ^**The dose equivalents for some "Large Joint" doses are derived from Klippel et al. (2001)^9 ^and Noerdlinger and Fadale (2001)^2^. A-C joint ranges are adapted from Saunders (Kenalog), and Tallia and Cardone (Depo-Medrol, Celestone Soluspan). Doses for other cortisone types not conisderered in these sources are derived from the dose equivalents shown above, with some corrections for inconsistencies in Noerdlinger and Fadale (2001) ^2^.

**^d ^**These values are estimated.

**Table 3:** Relative glucocorticoid potencies, and relative prednisone or glucocorticoid equivalents (per mg). Relative Glucocorticoid Potencies*, and Relative Prednisone or Glucocorticoid Equivalents For Intra-synovial Injections

**Author**	**Type of Equivalents**	**Celestone Soluspan**	**Decadron**	**Decadron L.A.**	**Kenalog**	**Aristospan**	**Depo-Medrol**	**Hydeltra T.B.A**	**Hydro-cortisone**
A. Relative Potencies (unit-less values) *									

Genovese (1998)^17^	Glucocorticoid Potency	25	25	25	5	5	5	4	1
Tallia and Cordone (2003)^3^	Glucocorticoid Potency	25	25	25	5	5	5	4	1
Greene (2001)^7^	Glucocorticoid Potency	25	-	-	5	5	4	4	1
Klippel et al. (2001)^9^	Glucocorticoid Potency	25	25	25	5	5	5	4	1
Axelrod (1976, 1979)^10,11^	Glucocorticoid Potency	25	30	-	5	5	5	4	1
Bird (2003)^37^	Glucocorticoid Potency	-	-	25	5	5	5	-	3.9
Walsh and Rogers (2004)^31^	Glucocorticoid Potency	25	-	-	5	-	5	4	1

**B. Prednisone Equivalence (in mg prednisone)**									

Owen (2001)^32^	Prednisone Equivalence	10	8	-	10	5	10	4	1
Wise (2005)^1^	Prednisone Equivalence	10	8	16	10	5	10	4	1

**C. Glucocorticoid Equivalence (relative mg values)**									

Axelrod (1976, 1979)^10,11^	Glucocorticoid Equivalence	0.6	0.75	-	4	4	4	5	20
Noerdlinger and Fadale (2001)^2^	Glucocorticoid Equivalence	0.6	0.75	-	4	4	4	5	20
Cush and Kavanaugh (2000)^6^	Glucocorticoid Equivalence	0.6	-	-	4	4	4	5	-

**Relative Potencies Used in Current Study (See Table 2):**		25	25	25	5	5	5	4	1

* The values listed for glucocorticoid ("cortisone") potencies are relative; hydrocortisone is arbitrarily assigned a value of 1. Axelrod (1976, 1979) ^10,11 ^is the only listed author that provides extensive sources for this information.

In **Part A **"Relative Potencies" should be read in the following manner: Celestone Soluspan is 25 times more potent then Hydrocortisone, and Kenalog is 5 times more potent than Hydrocortisone, and etc.

***Note***: Confusion often arises when making direct correlation between mg equivalence and relative potencies. They are typically inversley related. However, relative solubility variations can also influence these values in ways that are not yet fully understood.

The goal of this study is to evaluate current trends of injectable corticosteroid use among orthopaedic surgeons and selected non-surgeon sub-specialists and specialty physicians (rheumatologists, primary-care sports medicine physicians, and physical medicine and rehabilitation physicians) for injecting degenerative and overuse shoulder conditions in order to provide data that will ultimately be needed to establish uniform guidelines and identify potential knowledge deficiencies. We focused on these physicians in our tri-state referral area (Utah, Idaho, Wyoming; population = ~4.5 million people) in the western United States. We hypothesize that: **1) **there are significant differences in types and doses of corticosteroid and local anesthetic used for shoulder injections within and between surgical and non-surgical specialists who treat various painful shoulder conditions, and **2) **doses of corticosteroid and local anesthetic administered in shoulder injections often fall outside presently recommended ranges. Additional considerations included questions regarding: **1) **the use of fluorinated vs. non-fluorinated corticosteroids, **2) **the use of acetate vs. phosphate types, **3) **the rationale for using a particular corticosteroid, **4) **adjustments regarding patient age, and **5) **adjustments for special considerations including young athletes and diabetics.

## Methods

We conducted a survey by mail of orthopaedic surgeons (n = 163), rheumatologists (n = 24), and specialty physicians [PCSMs = primary-care sports medicine physicians, and PMRs = physical medicine and rehabilitation physicians ("physiatrists") (n = 77)] in Utah, Idaho, and Wyoming regarding their use of corticosteroid for painful shoulder conditions. These physicians (surgeons and non-surgeons) practice within the referral area of our orthopaedic shoulder-specialty practice in Salt Lake City, Utah, USA. The mailing addresses were obtained from membership directories of the American Academy of Orthopaedic Surgeons, the Utah, Idaho, and Wyoming Medical Associations, and regional telephone directories.

Each physician was sent a cover letter, which was attached to the survey, that explicitly stated that the survey was designed to obtain information regarding the corticosteroid and local anesthetic doses used for injecting painful degenerative and overuse conditions, strains (e.g., acromioclavicular joint), and related peri-scapular trigger points. Inflammatory conditions such as rheumatoid and lupus arthritis were expressly excluded. Trigger points were also defined as discrete, focal, hyperirritable foci located in taut peri-scapular musculature [14]. Each physician was asked 14 questions (see Appendix), including which types and amounts of corticosteroid(s) and local anesthetic(s) that they use when injecting five areas/diagnoses of the shoulder: the subacromial (S-A) bursa, acromioclavicular (A-C) joint, glenohumeral (G-H) joint, biceps tendon sheath, and peri-scapular trigger points. Other questions dealt with differences between acetate and phosphate corticosteroid types, fluorinated and non-fluorinated types, and whether or not the physicians used specific corticosteroid types when injecting some types of patients or patients with specific medical conditions (i.e., younger athletes or diabetics). Physicians were queried specifically about diabetic patients, and were asked to indicate any other patients or medical conditions for which they made modifications in corticosteroid types, or for which they chose not to administer these injections. The content of the questions were derived from a literature review of Cochrane databases[15] and various other sources, several of which are referenced in the body of the present study [2,16-18]. Most questions were posed in a close-ended format in accordance with Dillman (2000) for this form of behavioral survey [19]. Six weeks after the first mailing, a second mailing was made to non-responding physicians to increase the response rate. There were no inducements for returning the survey. Results were compiled four months after the initial mailing.

A subsequent analysis was conducted six to nine months after the initial mailing in order to assess possible differences between responders and non-responders in the two groups with less than 65% response rate (orthopaedic surgeons and PCSM/PMR physicians). This was accomplished by sending surveys to non-responders with a transmittal letter from the principal investigator that stated: **1**) the importance of the study, and **2**) that completion of their survey is essential for ensuring statistical reliability of the study. From these groups of non-responders, 11 additional surveys were obtained from orthopaedic surgeons and seven from the PCSM/PMR physicians. Results of these surveys from these 'late-responders' (from the third mailing) were compared to the entire group of 'early responders' (from the first two mailings) in each group.

It should be noted that Celestone Soluspan^® ^(Betamethasone sodium phosphate-Betamethasone acetate) was not commercially available in the U.S. at the time that the survey was administered. Some physicians, however, had this product in their clinics, and therefore they were still using it when they were surveyed. Finally, all dosages that are reported as "volumes" of corticosteroid (in cc) are normalized to 40 mg/ml of Depo-Medrol^® ^(Methylprednisolone acetate) (Table 2). Corticosteroid doses were considered "excessive" when they exceeded the ranges shown in table 2.

### Statistical analysis

For the observed sample sizes of n = 105 orthopaedic surgeons, n = 44 PCSMs/PMRs, and n = 20 rheumatologists, a post-hoc power analysis was conducted, based on a two-sided, alpha 0.05 comparison. For comparisons of continuous variables, the study had at least 95% power to detect a one standard deviation difference in means between any of the three groups. For a one-half standard deviation difference, there was 80% power for the orthopaedic surgeons vs. PCSMs/PMRs comparison, and approximately 50% for the other two comparisons. For comparisons of categorical variables, to detect an absolute difference between groups of 30% vs. 60%, in "yes" responses for example, the study had 90% power for the orthopaedic surgeons vs. PCSMs/PMRs comparison, 64% power for the orthopaedic surgeons vs. rheumatologists comparison, and 50% power for the PCSMs/PMRs vs. rheumatologist comparison. For a difference of 20% or smaller, no group comparison exceeded 57% power. Thus, the study was adequately powered to detect moderately large differences between means, but was inadequately powered to detect smaller, but reasonable group differences for categorical variables.

For pairwise comparisons of groups on continuous variables, such as corticosteroid doses and local anesthetic volumes, a two-sample t test was used. For pairwise comparisons of categorical variables, a chi-square test was used. Spearman correlation was used to test for an association between corticosteroid and local anesthetic volumes and an ordered categorical measurement of physician years of clinical practice, scored as (0–5 years = 1, 6–10 years = 2, 11–15 years = 3, 16–20 years = 4, >20 years = 5). Comparisons of early responders with late responders where similarly made. Given the already low power of the study, no adjustment was made for multiple comparisons. All statistical comparisons were made using Statview statistical software (SAS Institute, Cary, NC, USA). Significance was set at p < 0.05.

## Results

**Figure 1:**
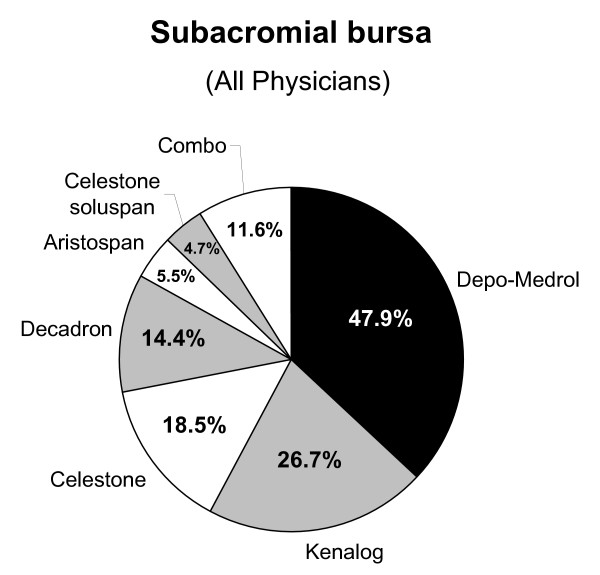
**Corticosteroid types used by responding physicians to inject the S-A bursa**. Data are shown as the percent of physicians using each corticosteroid type. Note that several physicians use one or two corticosteroids (but not in combination) for a given injection site (see text for details). This accounts for the cumulative percentage of >100% in these figures. These results are grossly similar for the G-H and A-C joints.

Of the 264 surveys that were mailed, 169 usable surveys were returned (64% overall response rate) from the early responders (data reported here are from early responders; see below for results of early vs. late responder analyses), including 105/163 orthopaedic surgeons (64% response rate), 44/77 PCSMs/PMRs (57% response rate), and 20/24 rheumatologists (83% response rate). Of the responding physicians, 150 (89%) treat painful shoulder conditions with corticosteroid injections; the most common locations being the S-A bursa (97%) and the A-C joint (83%). The type of corticosteroid and the percentage of physicians who inject the S-A bursa are shown in figure 1. These trends were commensurate with the other three shoulder regions, although fewer total physicians inject the G-H joint (76.4%), the biceps tendon sheath (67.3%), and peri-scapular trigger points (58.7%). Relative frequencies within each group of the two most commonly injected corticosteroids, or corticosteroid combinations, for the A-C and G-H joints, and S-A bursa are shown in Table 4.

**Table 4:** Frequencies of corticosteroid types used by responding physicians separated into each specialty group. Combo = corticosteroid combinations used. Contingency Table for Most Common Corticosteroids, or Corticosteroid Combinations

**Orthopaedic Surgeons**				
	**Depo-Medrol^®^**	**Kenalog^®^**	**Combo**	**TOTAL**

**A-C Joint**	43	18	9	**70**
**S-A Bursa**	35	20	13	**68**
**G-H Joint**	36	14	8	**58**

**TOTAL**	**114**	**52**	**30**	**196**

**PCSMs/PMRs**				

	**Depo-Medrol^®^**	**Kenalog^®^**	**Combo**	**TOTAL**

**A-C Joint**	17	8	0	**25**
**S-A Bursa**	15	12	2	**29**
**G-H Joint**	11	10	2	**23**

**TOTAL**	**43**	**30**	**4**	**77**

**Rheumatologists**				

	**Depo-Medrol^®^**	**Kenalog^®^**	**Combo**	**TOTAL**

**A-C Joint**	2	3	0	**5**
**S-A Bursa**	10	6	2	**18**
**G-H Joint**	7	4	1	**12**

**TOTAL**	**19**	**13**	**3**	**35**

**Combo **= combinations of two or more corticosteroids.

Data regarding gender and years-in-practice are summarized in Table 5. For all injected locations in the two groups with males and females (PCSMs/PMRs and rheumatologists), there were no significant male vs. female differences (all p values > 0.49) in corticosteroid doses and local anesthetic volumes. Using *combined *data from the three physician groups for each of the five injected locations, corticosteroid doses and local anesthetic volumes showed little or no correlation with years in practice (the absolute values of all ten r values are ≤ 0.238 with only one p value < 0.05). Analysis of each of the three physician groups yielded similar results for: 1) the orthopaedists for corticosteroid *and *local anesthetic injections (the absolute values of all ten r values are ≤ 0.206 and all p values ≥ 0.07); 2) the PCSMs/PMRs for local anesthetic volumes only (the absolute values of all five r values are < 0.210 and all p values > 0.3); and 3) the rheumatologists for local anesthetic volumes only (the absolute values of all five r values are < 0.340 and all p values > 0.4). In contrast, years-in-practice and corticosteroid doses showed significant (p ≤ 0.05), or tendencies toward significant (0.05 < p ≤ 0.12), inverse relationships in the: 1) PCSMs/PMRs, with these r values ranging from -0.327 (G-H joint, p = 0.12) to -0.466 (A-C joint, p = 0.04); and 2) rheumatologists, with these r values ranging from -0.533 (S-A bursa, p = 0.01) to -0.759 (trigger points, p = 0.05).

**Table 5:** Physician characteristics: gender and years-in-practice. Physician Characteristics (excluding late responders)

	Female (%)	Male (%)	Years in practice	% of total responding physicians per time interval	% of responding physicians who answered this question
**Orthopaedists**	0%	100%	0–5	7.7% (n = 7)	
			6–10	16.5% (n = 15)	
			11–15	20.9% (n = 19)	86.7%
			16–20	14.3% (n = 13)	
			>20	40.2% (n = 37)	

**PCSMs/PMRs**	24.4%	75.6%	0–5	16.2% (n = 6)	
			6–10	32.5% (n = 12)	
			11–15	21.6% (n = 8)	84.1%
			16–20	13.5% (n = 5)	
			>20	16.2% (n = 6)	

**Rheumatologists**	7.7%	92.3%	0–5	20% (n = 4)	
			6–10	20% (n = 4)	
			11–15	20% (n = 4)	100.0%
			16–20	15% (n = 3)	
			>20	25% (n = 5)	

Early-responder vs. late-responder analysis did not demonstrate significant differences (p > 0.1) within each of the two groups with response rates of <65% (orthopaedic surgeons and PCSMs/PMRs).

### Hypothesis 1

Although there is variability between specialties in the type of corticosteroid used for injecting painful shoulder conditions, there was no statistically significant difference in the doses used between the specialties for any particular joint injection (p > 0.3 for all inter-group comparisons for each injected location). This refutes the first facet of our first hypothesis. In contrast, the volumes of local anesthetic used showed greater variability (Table 6); orthopaedic surgeons used significantly greater volumes (p < 0.01) than PCSMs/PMRs and rheumatologists for the S-A bursa and G-H joint (and these latter two physician groups were not significantly different (p > 0.3)). This supports the second facet of our first hypothesis.

**Table 6:** Mean volumes [g/cc] and (standard deviation) of corticosteroid and local anesthetic. Mean volumes [cc] and (standard deviation) of cortisone and local anesthetic

	**Acromioclavicular (A-C) Joint**		**Subacromial (S-A) Bursa**		**Glenohumeral (G-H) Joint**	
			
	**Cortisone^a,b^**	**Local**	**Cortisone^a,b^**	**Local**	**Cortisone^a,b^**	**Local**
**Rheumatologists**	0.8 (0.3)	1.1 (0.5)	1.1 (0.5)	2.1 (2.1)	1.1 (0.5)	2.3 (1.5)
**Sports Medicine & Physiatrists (PCSMs/PMRs)**	0.8(0.5)	1.2 (0.6)	1.4 (0.7)	3.2 (1.9)	1.4 (0.7)	3.2 (1.9)
**Orthopaedic Surgeons**	0.9 (1.1)	1.5^c ^(1.4)	1.6 (2.1)	4.7^c ^(2.8)	1.6 (2.3)	4.7^c ^(2.6)

**^a ^**1 cc of "cortisone" (i.e., a corticosteroid of any given type) represents the equivalent of 40 mg of Depo-Medrol.

**^b ^**There are no significant differences in cortisone doses between the three physician groups.

**^c ^**When compared to each of the other groups, orthopaedic surgeons use significantly (p < 0.01) greater volumes of local anesthetic in each location. Rheumatologists and PCSMs/PMRs do not differ in anesthetic volumes (p > 0.3).

### Hypothesis 2

Among all of the specialties, the recommended corticosteroid dose ranges (shown in Table 2) were exceeded mostly in the A-C joint (orthopaedists 41%; rheumatologists 44%; PCSMs/PMRs 29%). Despite these exceeded recommended doses for the A-C joints, >98% were within recommendations for the S-A bursa and G-H joint. In these two locations, only a few orthopaedic surgeons exceeded these dose ranges (4% and 2%, respectively). Among those orthopaedic surgeons, two surgeons injected 1.5 times the highest recommended dose for the S-A bursa or G-H joint.

### Additional considerations

Injections in the biceps tendon sheath and the trigger points showed little to no variability among the specialties and are therefore given little mention in the discussion and figures. However, analysis of corticosteroid types used in these extra-articular locations showed significant inter-specialty differences in the use of fluorinated vs. non-fluorinated corticosteroids. Fluorinated corticosteroids (Table 2), which can be more deleterious to soft tissues (see discussion below), were used with the following frequencies: **Biceps sheath**: 17% rheumatologists, 8% PCSMs/PMRs, 37% orthopaedists; **Trigger points**: 10% rheumatologists, 5% PCSMs/PMRs, and 34% orthopaedists. In turn, the majority of physicians used non-fluorinated corticosteroids for each location.

The majority (85%) of all responding physicians use the same corticosteroid for each injection site. When asked for a rationale for using a particular corticosteroid, responses varied widely and included: long/short acting (30.3%), availability and/or force of habit (15.3%), faster (or slower) onset (12.2%), lower associated pain within 48 hours of the injection (9.1%), and solubility (8.3%).

Data regarding acetate vs. phosphate types of corticosteroids are summarized in Table 7. Among the physicians who used different types of corticosteroids depending on the condition being treated, acetate types (less soluble) were most commonly used for chronic conditions, including arthritis, bursitis, biceps tendinitis, impingement, and rotator cuff tendinitis. Phosphate types (more soluble) were used more frequently in acute conditions such as bursitis, muscle contusion, and adhesive capsulitis. More rheumatologists (80%) were aware that there are acetate and phosphate types of corticosteroids as compared to PCSMs/PMRs (76%) and orthopaedic surgeons (59%). However, relatively less rheumatologists (25%) than PCSMs/PMRs (32%) or orthopaedic surgeons (32%) knew that phosphate types are more soluble (Table 7).

**Table 7:** Physician responses to selected survey questions (acetate vs. phosphate, and diabetes). Acetate vs. Phosphate, & Diabetes, Contingency Tables

**Question #5a – Were you aware that there are acetate and phosphate types of corticosteroids?**				
	**Orthopaedists**	**PCSMs/PMRs**	**Rheumatologists**	**TOTAL**

**Yes**	55(59.1%)	28(76%)	16(80%)	**99**
**No**	36(38.7%)	9(24%)	4(20%)	**49**
**No Answer**	2(2.2%)	0(0%)	0(0%)	**2**

**TOTAL**	**93**	**37**	**20**	150*

**Question #5b – If yes, which type is more soluble?**				

	**Orthopaedists**	**PCSMs/PMRs**	**Rheumatologists**	**TOTAL**

**Phosphate**	30(32.2%)	12(32.4%)	5(25%)	**47**
**Acetate**	6(6.5%)	3(8.1%)	2(10%)	**11**
**Don't Know**	57(61.3%)	22(59.5)	13(65%)	**92**

**TOTAL**	**93**	**37**	**20**	150*

**Question #6 – Were you aware that acetate vs. phosphate corticosteroids may have different degrees of local and systemic absorption, and differences in duration of their anti-inflammatory affect?**				

	**Orthopaedists**	**PCSMs/PMRs**	**Rheumatologists**	**TOTAL**

**Yes**	40(43%)	19(51.4%)	10(50%)	**69**
**No**	51(54.8%)	16(43.2%)	10(50%)	**77**
**No Answer**	2(2.2%)	2(5.4%)	0(0%)	**4**

**TOTAL**	**93**	**37**	**20**	150*

**Question #7 – Do you ever use acetate-type corticosteroids (instead of phosphate-types) for treating specific shoulder conditions?**				

	**Orthopaedists**	**PCSMs/PMRs**	**Rheumatologists**	**TOTAL**

**Yes**	35(37.6%)	13(35.1%)	3(15%)	**51**
**No**	49(52.7)	21(56.8%)	13(65%)	**83**
**No Answer**	9(9.7%)	3(8.1%)	4(20%)	**16**

**TOTAL**	**93**	**37**	**20**	150*

**Question #8 – Do you ever use phosphate-type corticosteroids (instead of acetate-types) for treating specific shoulder conditions?**				

	**Orthopaedists**	**PCSMs/PMRs**	**Rheumatologists**	**TOTAL**

**Yes**	21(22.6%)	9(24.3%)	3(15%)	**33**
**No**	66(71%)	26(70.3%)	17(85%)	**109**
**No Answer**	6(6.4%)	2(5.4%)	0(0%)	**8**

**TOTAL**	**93**	**37**	**20**	150*

**Question #9 – Do you use a different type of corticosteroids for injecting the shoulder region of diabetic patients?**				

	**Orthopaedists**	**PCSMs/PMRs**	**Rheumatologists**	**TOTAL**

**Yes**	8(8.6%)	5(13.5%)	2(10%)	**15**
**No**	85(91.4%)	31(83.8%)	18(90%)	**134**
**No Answer**	0(0%)	1(2.7%)	0(0%)	**1**

**TOTAL**	**93**	**37**	**20**	150*

* The total number of physicians represents the 150 of the 169 early responders who actually treat painful shoulder conditions with corticosteroids (93 orthopaedic surgeons, 37 PCSMs/PMRs, and 20 rheumatologists.)

**Figure 2:**
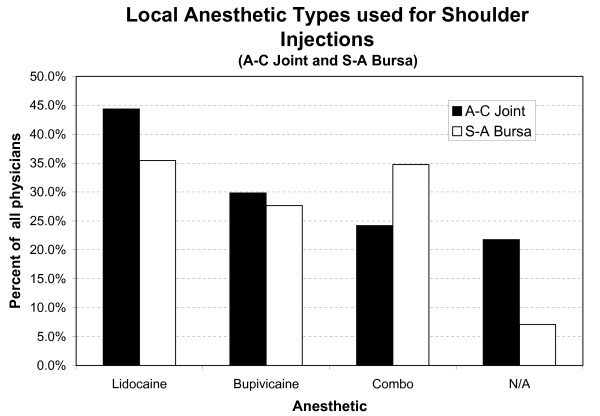
**Types of local anesthetics used by all responding physicians**. S-A bursa and G-H data are similar. **N/A **= not applicable (i.e., physicians who inject painful shoulder conditions as part of their practice but who do not inject the joint indicated); **Combo **= combination of Lidocaine and Bupivicaine. Bupivicaine has significantly prolonged onset of anesthesia (~2–10 minutes) when compared to lidocaine (seconds to minutes) (package product information, Abbott Laboratories, North Chicago, IL, USA).

When asked about types/volumes of local anesthetic that they administered with corticosteroid injections, 92 physicians (74.2%) use Lidocaine (Xylocaine^®^) or Bupivicaine (Marcaine^®^) when injecting the A-C joint. The type of local anesthetic and the percentage of physicians who inject them are shown in figure 2. Volumes of local anesthetic vary considerably for all shoulder conditions, and most significantly in the S-A bursa where the range is 0.5 cc to 10 cc (Table 6).

Only 15 physicians (10.0%) reported any change in treatment for diabetic patients including two (1.3%) who do not inject them. The number and (percentage) of physicians in each group who did alter their practice for diabetics are as follows: 8/93(8.6%) orthopaedists; 5/37(13.5%) PCSMs/PMRs; 2/20(10%) rheumatologists (Table 7 bottom). Seven physicians (4.7%, three orthopaedists, three PCSMs/PMRs, and one rheumatologist) simply inject less corticosteroid for diabetics, and only one (0.7%, an orthopaedist) physician explicitly stated that he had these patients closely monitor their blood glucose levels for one week after the injection. Claiming fewer systemic effects, two physicians (1.3%, one orthopaedist and one rheumatologist) use phosphate derivative corticosteroids for diabetic patients. None of the responding physicians varied their corticosteroid type or volume for patients with other (non-diabetic) medical conditions. Only six physicians (4.0%, three orthopaedists, one PCSMs/PMRs, and two rheumatologists) used a different corticosteroid type or dose for young athletes compared to less active middle-aged patients. Less than 2% of all survey respondents (two orthopaedists) injected painful (non-arthritic) A-C and G-H joints in young patients or athletes.

## Discussion

Results of our survey show that, on average, the dose equivalents of corticosteroids used for injecting the various shoulder regions do not differ significantly between orthopaedic surgeons, rheumatologists, and specialty physicians (PCSMs = primary-care sports medicine physicians, and PMRs = physical medicine and rehabilitation physicians). This contradicts one facet of our first hypothesis predicting that significant differences would be found in corticosteroid doses between these groups. Results of our retrospective sample-size and power calculations (see Methods) also suggest that these small differences are not the result of statistical error. Although table 2 provides general guidelines for recommended doses and dose equivalents for common injectable corticosteroids, for most shoulder conditions it is not clear how much corticosteroid/anesthetic is appropriate since uniform guidelines for these injections are not firmly established. This is evident in the broad variations in dose ranges recommended in recent literature for commonly injected locations/conditions (Table 1). The present study shows that there is a wide variety of corticosteroid types used for each injected location for degenerative and/or overuse conditions and reported doses show a broad range within each specialty group. However, only 3–4% of surgeons and PCSMs/PMRs use doses exceeding the "recommended" range for the G-H joint and the S-A bursa. In contrast, 39% of all physicians (41% orthopaedic surgeons, 44% rheumatologists, 29% PCSMs/PMRs) exceeded the recommended dose range for the A-C joint. It is possible that the most commonly used corticosteroids, Depo-Medrol^® ^and Kenalog^®^, reflect their comparative costs in U.S. dollars (e.g., $5.00 for 40 mg Depo-Medrol^®^; $6.90 for 40 mg Kenalog^®^; $6.80 for 6 mg Celestone Soluspan^®^; $6.00 for 20 mg Aristospan^®^; $7.60 for 4 mg Decadron^®^).

In contrast to data for corticosteroid doses, the volumes of local anesthetic used for injecting these painful shoulder conditions varies significantly between the physician groups, corroborating this facet of our first hypothesis. For example, in the perspective of our shoulder specialty practice the amount of local anesthetic typically used by PCSMs/PMRs and rheumatologists seems insufficient, especially for the S-A bursa. This view is based on our use of volumes commensurate with Neer's "impingement test", where 10 cc of local anesthetic (1% Lidocaine) is used to adequately infuse the S-A bursa [20]. Pain relief with provocative maneuvers of the shoulder, indicative of a positive test, is useful for establishing a S-A impingement lesion as the cause of pain [21]. However, most of the physicians (predominantly non-surgeons) used less than half of the 10 cc volume described by Neer. We speculate that the relatively smaller volume of anesthetic (5 cc) used by Kirkley et al. (2002) [22] to rigorously evaluate the Neer impingement test might help to explain why these investigators concluded that this test might be insufficient for predicting the success of surgery [23]. Recent published recommendations for total dose/volume of corticosteroid/anesthetic solutions also typically fall 3–6 cc short of the 11–12 cc that would be administered when corticosteroid is added to the volume of local anesthetic (10 cc) used in the Neer impingement test [3,4,24,25]. Infusing corticosteroid with such 'large' volumes of local anesthetic may also enhance the distribution of corticosteroid throughout the bursa, especially when inflammatory bursa separations or compartmentalization exists [26]. In our shoulder-specialty practice we consider 'larger' volumes of local anesthetic, such as those described by Neer [20], as an essential component for these injections [23]. In turn, results of the present study showing variations between specialists in anesthetic doses suggest that surgeons (who use significantly larger anesthetic volumes) emphasize determining the percentage of pain attributable to the injected region as a tool for surgical planning. However, it is also plausible that, when compared to surgeons, non-surgeons inject lower volumes of local anesthetics because they have more profound knowledge of potential cardiovascular side effects resulting from the inadvertent intra-vascular injection of these agents. For example, there are reports of hypotension, bradycardia, and cardiac arrest resulting from the intra-vascular injection of Bupivicaine and Lidocaine [27-31].

Effective uniform guidelines for corticosteroid injections must consider the possibility that doses exceeding recommended ranges could cause additional systemic and local side effects. Deleterious consequences and other sequelae of corticosteroid injections are frequently a result of chronic use, and may be more prevalent if the corticosteroid spreads to adjacent tissues [8,32,33]. Consideration for systemic consequences are also important, especially for patients with diseases that cause immunosupression (e.g., diabetes mellitus and rheumatoid arthritis) [4,16,17,34,35]. Systemic dissemination of corticosteroid can occur after local injection, further reducing endogenous cortisol levels and exacerbating immunosupression [32,36,37]. In this perspective it is interesting that only 10% (15/150) of the survey respondents (two of these were rheumatologists) report any modifications in corticosteroid dose or type, or adjustments in glucose monitoring for diabetic patients. In our clinical practice we have our diabetic patients monitor their glucose levels more closely (i.e., every six hours), and have them adjust their medication doses for one-to-two weeks after receiving the corticosteroid injection. Although we have observed that insulin-dependent and non-insulin-dependent diabetic patients can have broad fluctuations in finger-stick blood glucose measurements (exceeding 400 mg/dL) for several days following an S-A or G-H injection, published data on this issue are contradictory [38,39]. Further studies are therefore warranted for determining whether adjustments of corticosteroid types, doses, and dose intervals are necessary when injecting diabetic patients.

It has been noted in rheumatology literature that fluorinated corticosteroids (e.g., Celestone Phosphate^®^, Celestone Soluspan^®^, Decadron^®^, Kenalog^®^, and Aristospan^®^) (Table 2), when compared to non-fluorinated corticosteroids (e.g., Hydeltrasol^®^, Depo-Medrol^®^, Hydeltra-TBA^®^, and Hydrocorticone^®^), are more highly associated with tendon rupture and subcutaneous atrophy [8]. For this reason, Moore suggests avoiding the use of fluorinated corticosteroids for extraarticular injections [8]. This is because soft tissue injections of these agents can cause significant atrophy of collagenous tissue that can lead to ligament or tendon rupture or subcutaneous calcification and/or atrophy (the atrophy may disappear in 2 to 3 years). Results of our survey suggest that few physicians, especially orthopaedic surgeons, recognize or accept the putative importance of this issue for selecting a particular corticosteroid type for extra-articular injections. This seems evident in results showing that 17% of rheumatologists, 8% of PCSMs/PMRs, and 37% of orthopaedists typically used fluorinated corticosteroids for injecting the biceps tendon sheath, and 10% of rheumatologists, 5% of PCSMs/PMRs, and 34% of orthopaedists use fluorinated corticosteroids for injecting trigger points.

Our survey also queried physicians regarding whether or not they made adjustments in corticosteroid dosing based on patient age. The only notable finding was that less than 1% of all survey respondents injected painful (non-arthritic) A-C and G-H joints in young patients or athletes. This probably reflects the philosophy, common in sports medicine literature, that performing these injections could exacerbate the injury because pain-related perception, which helps to 'protect' the joint by limiting its use, is diminished [32,40-42]. Similarly, it has been suggested that pain relief from corticosteroid injections into *degenerative *joints can accelerate the degenerative process by allowing increased use (and further deterioration) of the joint [40,43]. Although this idea is typically applied to weight-bearing joints, it might also apply to the A-C and G-H joints.

The possibility that exceeding dose ranges for intra-articular injections (as reported by ~37% survey respondents for the A-C joint) can have deleterious effects on articular cartilage is, however, suggested by indirect or anecdotal evidence. For example, injections of high dose (50–100 mg) betamethasone acetate once a week for two-to-four weeks into the knees of four-to-six month old rabbits caused distortion of articular chondrocyte shape and loss of cell organelles, thus affecting the normal production of collagen fibers responsible for articular cartilage strength [44,45]. Subtle cell distortion was detected after only two doses (necropsy at 28–42 days post-injection). These and other studies showing deleterious effects in growing animals [32], however, might not be applicable when 'high' doses are injected into *degenerative *joints in humans. For example, Hollander and co-workers have shown little evidence of morbidity of intra-articular corticosteroid injections that have been used for many years as a primary treatment modality for osteoarthritis of the knee [46,47]. Owen (2001) [32] also notes that the concept of glucocorticoid arthropathy is based largely on anecdotal case reports and subprimate animal studies. By contrast, studies of primate models have shown no long-term adverse effect on cartilage [48]. There is also a well documented report of a 51-year-old women who received 100 glucocorticoid injections [using Hydeltra TBA, Celestone Soluspan, or Kenalog] into each knee during a span of 10 years with no deleterious effects seen on knee radiographs taken before and after these treatments [49]. Additionally, Balch et al. (1977) studied knee radiographs of 65 patients with osteoarthritis or rheumatoid arthritis who received repeated injections extending from four to 15 years [50]. The radiographs of 15 patients showed no deterioration, 38 showed minimal to moderate deterioration, 10 showed marked deterioration, and only two showed gross deterioration. Although twelve of these patients (12/65, 18.5%) appear to have clearly developed what some might call "steroid arthropathy", the results in the majority of these patients do not support the contention that repeated intra-articular injections of corticosteroids into arthritic knees would inevitably lead to rapid joint destruction.

Even in view of these data and observations – which are now early 20 years old – deleterious consequences of intra-articular injections shown in studies of sub-primate animals appear to have swayed most experts toward warning that similar consequences might also occur in degenerative joints in humans. As noted, our survey did not evaluate the dosing intervals typically used by the physicians in each group. Therefore, it is not clear if 'excessive' doses – irrespective of dose intervals – used by some of our surveyed physicians could enhance the progression of arthritis.

Results of correlation analyses showed a tendency toward reduced corticosteroid doses with years-in-practice in the PCSMs/PMRs and rheumatologist groups. The strongest relationship was found in the rheumatologists who showed reduced corticosteroid doses for periscapular trigger points (r = -0.759, p = 0.05). Additional studies would be required to first corroborate these findings and, second, to determine if they reflect temporal differences in training or a trend that occurs with clinical experience. For example, it would be interesting to determine if this trend in trigger points reflects clinical experience that these rheumatologists might have with subcutaneous atrophy caused by injecting corticosteroid injections.

Limitations of our study include its regional focus on physicians in our tri-state referral area of the western United States. Although these results may not apply to broader geographical regions of the United States, in the context of our literature review they do illustrate the lack of uniform injection guidelines in the general literature. Furthermore, our data might not reflect the injection practices of surgeon and non-surgeon peers outside of the United States. Another limitation included the relatively lower response rate of PCSMs/PMRs (56%), when compared to the higher response rates of rheumatologists and orthopaedic surgeons (83% and 63% respectively). Early-responder vs. late-responder analyses did not demonstrate significant differences within the two groups with response rates of <65%. However, given that there were only 11 and 7 responses in these respective groups from the later mailing, it is unlikely that the data in this context is suitably powered for finding any statistically significant differences. Although these data do not rigorously rule-out early-responder vs. late-responder bias, they tend to reduce this possibility as a confounding factor in generalizing the results as representative of the injection practices of the physician groups that were surveyed. Therefore, the current study provides useful baseline data for guiding future studies of physicians in larger geographical areas. In addition to aiding in establishing uniform injection guidelines, our results also indicate the clear need for advanced education with regards to some aspects of the use of corticosteroid/anesthetic injections.

Future surveys that are designed for broader geographical distribution should also consider additional medical conditions or concerns that were not explicitly addressed in the present study. For example, further clarification of clinical practice of the surgeons and non-surgeon groups should include questions regarding whether or not they would consider injecting, or modifying injections, in patients taking anticoagulant medications. For example, although many physicians might consider the concomitant use of Warfarin as a contraindication of these injections, it is not know exactly how prevalent this opinion is and/or if this opinion varies in cases where patients are taking other types of anticoagulant medications (e.g., aspirin or clopidogrel bisulfate).

## Conclusion

Variations shown in this study between specialists in anesthetic doses suggest that surgeons (who use significantly larger anesthetic volumes) emphasize determining the percentage of pain attributable to the injected region. Alternatively, this might reflect a more profound knowledge that non-surgeons specialists have of the potentially adverse cardiovascular effects of these agents. Although the ranges of corticosteroid doses for each injection location were broad, the mean doses did not differ significantly between the groups. Nearly 85% of the respondents use the same corticosteroid for all injections, and fewer than 10% make adjustments for diabetic patients. Rheumatologists seemed more cognizant of the clinical importance of distinguishing corticosteroids based on fluorination vs. non-fluorination, and acetate vs. phosphate composition. These results help provide useful information for future studies aimed at establishing uniform guidelines for treating painful shoulder conditions with injectable corticosteroids, and for identifying knowledge deficiencies that warrant advanced education. Uniform guidelines are important for: **1) **maximizing pain relief in patients with chronic and acute painful shoulder conditions, **2) **improving communication between surgeon and non-surgeon sub-specialists and general health-care providers, **3) **eliminating potentially excessive corticosteroid exposure and adverse events, and **4) **enhancing the localization of source(s) of pain for diagnostic/prognostic purposes.

## Competing interests

This study was supported by an unrestricted research grant from Pharmacia and Upjohn Companies, Inc.

## Authors' contributions

JS conceived the study, and participated in its design and coordination and helped to draft the manuscript. KH assisted in the creation and design of the study and sent out the initial surveys. TP assisted in the non-responders survey, performed the statistical analyses and helped draft and finalize the manuscript. All authors read and approved the final manuscript.

## Pre-publication history

The pre-publication history for this paper can be accessed here:



## Supplementary Material

Additional file 1 Survey Appendix. This is a copy of the survey that was sent out to each physician. Click here for file
