# Supplementary material for: Variations in corticosteroid/anesthetic injections for painful shoulder conditions: comparisons among orthopaedic surgeons, rheumatologists, and physical medicine and primary-care physicians
Source: BMC Musculoskelet Disord. 2007 Jul 6;8:63. doi: 10.1186/1471-2474-8-63 (PMC1950874; doi:10.1186/1471-2474-8-63)
Supplement: Additional file 1 — Survey Appendix. This is a copy of the survey that was sent out to each physician. [file 1471-2474-8-63-S1.pdf]

For this survey, refer to the enclosed table for corticosteroid types.

As noted in the cover letter, these questions refer to conditions that include injecting painful degenerative and overuse conditions, strains (e.g., acromioclavicular joint), or peri-scapular trigger points.

1. Do you use corticosteroid injections for treating painful shoulder conditions? Yes No

If yes, please fill out the remainder of the survey.
If no, please state why and return the survey. ____________________

____

2. Which types (and how much volume) corticosteroid do you typically use for the:

	Type of Corticosteroid(s) and strength (mg/ml) (see attached table for types)	Volume of Cortisone (cc) (please circle one)
Acromioclavicular joint	____________	.25 .50 .75 1.0 1.25 1.50 1.75 2.0 Other:___
Subacromial bursa	____________	.25 .50 .75 1.0 1.25 1.50 1.75 2.0 Other:___
Glenohumeral joint	____________	.25 .50 .75 1.0 1.25 1.50 1.75 2.0 Other:___
Biceps tendon sheath	____________	.25 .50 .75 1.0 1.25 1.50 1.75 2.0 Other:___
Trigger points	____________	.25 .50 .75 1.0 1.25 1.50 1.75 2.0 Other:___

3. If you use different types of corticosteroid for the different shoulder locations, please explain your rationale in choosing a specific corticosteroid for a particular area of the shoulder.

____

____

____

4. Which type (and how much volume) of local anesthetic do you typically use for the:

	Type of local anesthetic (please circle one or both if combo)		Volume of anesthetic (cc) (please circle one)
Acromioclavicular joint:	Lidocaine	Bupivicaine	1 2 3 4 5 6 7 8 9 10 Other:___
Subacromial bursa:	Lidocaine	Bupivicaine	1 2 3 4 5 6 7 8 9 10 Other:___
Glenohumeral joint:	Lidocaine	Bupivicaine	1 2 3 4 5 6 7 8 9 10 Other:___
Biceps tendon sheath:	Lidocaine	Bupivicaine	1 2 3 4 5 6 7 8 9 10 Other:___
Trigger points:	Lidocaine	Bupivicaine	1 2 3 4 5 6 7 8 9 10 Other:___

5. What do you think are the advantages of the corticosteroid(s) that you use?

	Corticosteroid Type	Advantages
1st Choice	____________	____________
2nd Choice	____________	____________

6. **Were you aware that there are acetate and phosphate types of corticosteroids?**

☐ Yes ☐ No

If yes, which type is more soluble?

☐ Acetate ☐ Phosphate ☐ Don't Know

7. **Were you aware that acetate <u>vs.</u> phosphate corticosteroids may have different degrees of local and systemic absorption, and differences in duration of their anti-inflammatory affect?**

☐ Yes ☐ No

8. **Do you ever use acetate-type corticosteroids (instead of phosphate types) for treating specific shoulder conditions? ***

☐ Yes ☐ No

If yes, what are these conditions or situations? ______________________________

What is your rational for using <u>acetate</u>-type corticosteroids for each of these conditions or situations?

____

____

9. **Do you ever use phosphate-type corticosteroids (instead of acetate types) for treating specific shoulder conditions? ***

☐ Yes ☐ No

If yes, what are these conditions or situations? ______________________________

What is your rational for using <u>phosphate</u>-type corticosteroids for each of these conditions or situations?

____

____

10. **Compared to the typical corticosteroid that you use for injecting the subacromial bursa, do you use a different type of corticosteroid for injecting the shoulder region of <u>diabetic</u> patients?**

☐ Yes ☐ No

If yes, which type of corticosteroid do you use and why? ___________________________

____

____

---

* For the purpose of this survey, "shoulder region" or "shoulder condition" refers to the acromioclavicular joint, subacromial bursa, glenohumeral joint and/or biceps tendon sheath. Additional "conditions" may include: frozen shoulder syndrome, scapulo-thoracic bursitis, and trigger points.

11. Compared to the typical corticosteroid that you use for injecting the subacromial bursa, do you use a different type of corticosteroid for injecting the shoulder region of patients with other specific (non-diabetic) medical conditions?

☐ Yes ☐ No

If yes, what are these conditions or situations? ____________________________________

____

____

12. Do you use a different type of corticosteroid for young athletes (<30 years) vs. middle-aged laborers?

☐ Yes ☐ No

If yes, which type of corticosteroid?

Young Athlete: __________________ Middle-age: ___________________

13. How many years have you been in the practice of medicine or surgery?

☐ 0-5 ☐ 6-10 ☐ 11-15 ☐ 16-20 ☐ >20

14. Your gender and age are:

☐ Male ☐ Female

Age: ____________

Table

Common Injectable Corticosteroids

Generic Name	Trade Name	Strength (mg/ml)
Betamethasone Sodium Phosphate	Celestone	3
Betamethasone sodium phosphate-betamethasone acetate	Celestone-Soluspan	4
Dexamethasone acetate	Decadron-LA	8
Dexamethasone sodium phosphate	Decadron	4
Hydrocortisone acetate	Hydrocorticone	25
Methylprednisolone acetate	Depo-Medrol	20, 40, 80
Prednisolone sodium phosphate	Hydeltrasol	20
Prednisolone tebutate	Hydeltrasol-TBA	20
Triamcinolone acetonide	Kenalog	10, 40
Triamcinolone diacetonide	Aristospan forte	25, 40
Triamcinolone hexacetonide	Aristospan	20
